# Characteristics and prognostic analysis of *Pneumocystis jirovecii* pneumonia in connective tissue diseases patients with interstitial lung disease: a retrospective study

**DOI:** 10.1007/s10067-025-07392-1

**Published:** 2025-03-06

**Authors:** Yujie Shi, Ruxuan Chen, Hongli Sun, Kai Xu, Mengqi Wang, Zhiyi Li, Chi Shao, Hui Huang

**Affiliations:** 1 Department of Pulmonary and Critical Care Medicine, Dongcheng District, Peking Union Medical College Hospital, Chinese Academy of Medical Sciences & Peking Union Medical College, #1 Shuaifuyuan Street, Beijing, China; 2 Department of Clinical Laboratory, Dongcheng District, Peking Union Medical College Hospital, Chinese Academy of Medical Sciences & Peking Union Medical College, #1 Shuaifuyuan Street, Beijing, China; 3 Radiological Department, Dongcheng District, Peking Union Medical College Hospital, Chinese Academy of Medical Sciences & Peking Union Medical College, #1 Shuaifuyuan Street, Beijing, China

**Keywords:** Characteristics, Connective tissue diseases, Interstitial lung disease, *Pneumocystis jirovecii* pneumonia, Prognosis

## Abstract

**Introduction:**

*Pneumocystis jirovecii* pneumonia (PJP) is a common opportunistic infection. With the wide application of glucocorticosteroids and immunosuppressants, the incidence and mortality rates of PJP in connective tissue disease (CTD) patients with interstitial lung disease (ILD) are increasing.

**Methods:**

We retrospectively enrolled consecutive CTD-ILD patients with PJP in our center between January 2014 and December 2022. Cox regression models were constructed to explore prognostic factors in CTD-ILD-PJP patients.

**Results:**

There were 159 CTD-ILD patients [60 (51, 68) years, 61.0% female] with PJP, 78 (49.1%) of whom died. Compared with those in the CTD-non-ILD-PJP group, there were more pneumomediastinum cases (16.4% vs. 6.7%, *p* = 0.030) and significantly higher all-cause mortality rates (49.1% vs. 33.7%, *p* = 0.019) in the CTD-ILD-PJP group. Multivariate analysis indicated that IIM (HR = 2.635, 95% CI: 1.383–5.019), pneumomediastinum (HR = 2.877, 95% CI: 1.483–5.582), oral candidiasis infection (HR = 2.596, 95% CI: 1.229–5.483), aspergilli infection (HR = 2.886, 95% CI: 1.412–5.900), and lower minimal albumin (Alb) (HR = 0.872, 95% CI: 0.819–0.927) were independent risk factors associated with poor survival in CTD-ILD-PJP patients.

**Conclusions:**

CTD-ILD-PJP patients were mainly middle-aged females and had higher mortality rates than CTD-PJP patients without ILD. IIM, pneumomediastinum, oral candidiasis infection, aspergilli infection, and lower minimal Alb were independent risk factors associated with poor survival in CTD-ILD-PJP patients.

**Table:** 

**Key Points** • *CTD-ILD-PJP patients had higher mortality rates than CTD-PJP patients without ILD.* • *IIM, pneumomediastinum, oral candidiasis infection, aspergilli infection, and lower minimal Alb were independent survival risk factors in CTD-ILD-PJP patients.* • *The study explored susceptibility and prognostic risk factors of CTD-ILD-PJP patients, to reduce the incidence and mortality.*

**Supplementary information:**

The online version contains supplementary material available at 10.1007/s10067-025-07392-1.

## Introduction

*Pneumocystis jirovecii* pneumonia (PJP) is a common opportunistic pulmonary infection. With the development of anti-human immunodeficiency virus (HIV) drugs and the application of standardized PJP management and prevention strategies, the incidence rate of PJP in HIV patients has gradually decreased. However, compared with HIV patients, PJP in non-HIV immunocompromised patients usually has a subtle onset but can rapidly progress in a short term and lead to respiratory failure [1, 2].

Glucocorticoids and/or immunosuppressants are widely used to treat connective tissue diseases (CTDs) and CTD-related interstitial lung disease (CTD-ILD), resulting in patients with CTD-ILD becoming more vulnerable to PJP. Recent studies have shown that CTD-PJP patients had the most severe cases in non-HIV patients [3]. And combined with underlying lung diseases, including ILD, was one of the risk factors for poor prognosis in CTD-PJP patients [4, 5]. Previous glucocorticoid therapy and decreased CD4^+^ T cell count might be risk factors for susceptibility to PJP in CTD-ILD patients [6]. However, few clinical studies have explored the characteristics and prognostic factors of PJP in CTD-ILD patients. Therefore, we conducted a retrospective analysis of CTD-ILD-PJP patients to explore their clinical characteristics and factors with prognostic value, which may assist in the early identification and diagnosis of PJP infection in CTD-ILD patients and improve their prognosis. First, we hypothesize that CTD patients with underlying ILD would have a higher risk of mortality after PJP infection compared to CTD patients without ILD. We further propose that mortality in CTD-ILD-PJP patients might be independently associated with different underlying CTD, treatment with immunosuppressive therapy status, co-infection status, etc.

## Materials and methods

### Patients

All enrolled patients who were discharged with diagnosis of PJP and CTD concurrent with ILD from January 2014 to December 2022 at Peking Union Medical College Hospital were screened for study eligibility using the hospital’s electronic medical records system. During the screening process, patients with any of the following characteristics were excluded from our study: (1) were younger than 18 years old; (2) had a positive HIV test; (3) had malignancies; and (4) did not have chest computed tomography (CT) imaging data in the hospital records.

**Fig. 1:**
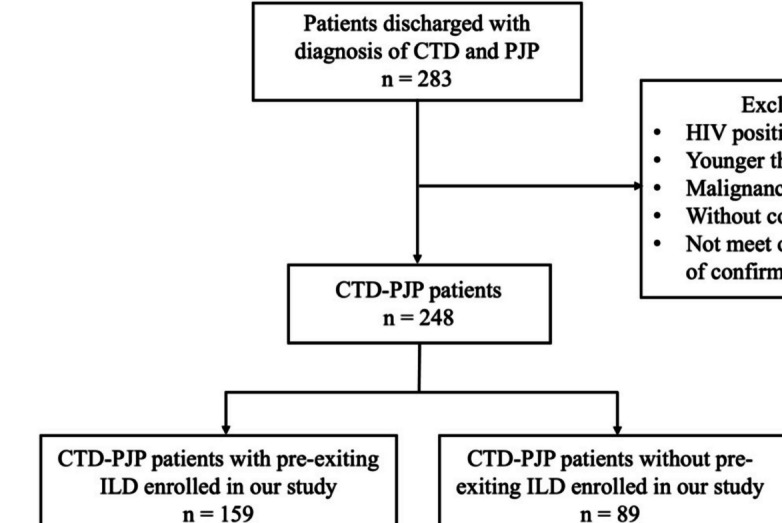
The study flow chart. CTD, connective tissue diseases; PJP, *Pneumocystis jirovecii* pneumonia; HIV, human immunodeficiency virus; ILD, interstitial lung disease

According to the inclusion and exclusion criteria, 159 hospitalized CTD-ILD patients with PJP were enrolled in our study. The study flow chart is shown in Fig. 1. The complete medical records and radiological imaging data of the patients were retrospectively reviewed using the hospital’s electronic medical records system. The patients were followed up from the day that the diagnosis of PJP was confirmed. After discharge, patients were followed every 1 to 6 months, depending on disease activity and severity. The final follow-up point was December 30, 2023. The follow-up information was obtained through outpatient records or telephone conversations. The following information was reviewed and analyzed: age, sex, clinical manifestations, serological results, chest high-resolution CT (HRCT) findings, treatments, and outcomes.

### Definitions

Diagnoses of CTDs were coincided with their respective classification criteria [7–12].

ILD was defined by the presence of hallmark manifestations on chest HRCT, compatible clinical manifestation, and sometimes pathological information. The HRCT were reviewed by two pulmonologists (C.S. and H.H.) and one radiologist (K.X.).

Diagnostic criteria of confirmed PJP were defined as follows: (1) new presence of relevant respiratory manifestations (cough, dyspnea, sputum, and/or hypoxia) or fever (temperature ≥ 37.3 ℃); (2) new presence of pulmonary shadows (ground-glass opacities, consolidation, or patchy shadows) that were identified by chest CT; (3) Pneumocystis DNA fragments were confirmed by polymerase chain reaction or metagenomic next-generation sequencing, and/or Pneumocystis asci were observed at direct microscopic examination with Grocott’s methenamine silver stain in the respiratory samples, including qualified sputum specimens, aspirates, or bronchoalveolar lavage fluid.

Respiratory failure was defined as a room air pulse oxygenation of < 90%, a room air arterial partial pressure oxygen level of < 60 mmHg (1 mmHg = 0.133 kPa), or the oxygenation index under oxygen inhalation is less than 300 mmHg.

Cytomegalovirus (CMV) viremia was defined as peripheral blood CMV-DNA > 500 copies.

Hospital-acquired pneumonia (HAP) in this study was defined as patients admitted to the hospital without infection or the incubation period, but developed pneumonia caused by bacteria in the hospital 48 h after admission.

This study was approved by the Ethics Committee of Peking Union Medical College Hospital (K5596) in accordance with the Declaration of Helsinki (approval date: March 29, 2024). Our study was performed using anonymized health care data. The written informed consent from each patient was waived as this study met the Peking Union Medical College Hospital institutional review board’s minimal risk waiver criteria.

### Statistical analysis

All the data were analyzed using the IBM SPSS Statistics version 27.0 software package (IBM Corporation, North Castle Drive, MD-NC119, Armonk, NY 10504–1785, USA). The quantitative variables were presented as the means ± standard deviations (SD) or medians (interquartile ranges [IQRs]), and the categorical variables were presented as frequencies and percentages. We conducted comparison between CTD-PJP patients with and without ILD, as well as the survival and non-survival groups of CTD-ILD-PJP patients, using t test, analysis of variance or rank sum test for quantitative variables, and the chi-square test for categorical variables. And Kaplan–Meier survival curves were used to compare mortality of different groups. Cox regression models were used to identify factors associated with prognosis for CTD-ILD-PJP patients. We defined death as the outcome variable, and survival time was defined as the time from the diagnosis of PJP to death or the final follow-up point. In univariate Cox regression analysis, Covariates included baseline characteristics, primary treatment for CTDs, co-infection status, chest CT features and treatment for PJP. Then the statistically significant variables selected via univariate analysis were finally assessed by multivariate analysis through forward stepwise regression method. Based on the Schoenfeld formula, assuming a target HR = 2.0, α = 0.05 (two-sided), statistical power of 80%, and an exposure group proportion of 50%, the required number of endpoint events is calculated to be 66. And 78 endpoint events were observed in the study, which met the statistical power requirements. The difference was statistically significant when *p* < 0.05.

## Results

### General characteristics of CTD-ILD-PJP patients

**Fig. 2:**
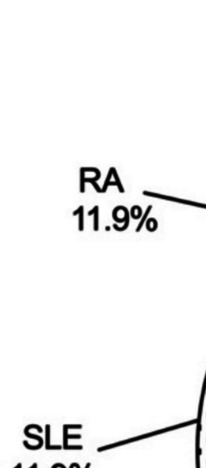
The underlying CTDs of enrolled 159 CTD-ILD-PJP patients. IIM, idiopathic inflammatory myopathy; SLE, systemic lupus erythematosus; RA, rheumatoid arthritis; SS, Sjogren's syndrome; SSc, systemic sclerosis; CTD, connective tissue diseases; ILD, interstitial lung disease

One hundred fifty-nine patients that were diagnosed with CTD-ILD-PJP were enrolled in our study after a detailed medical record review. Sixty-two patients were male (39.0%), 97 patients were female (61.0%), and the mean age was 60 (51, 68) years. The distribution of underlying CTDs of CTD-ILD-PJP patients mainly included systemic vasculitides (35.8%), idiopathic inflammatory myopathy (IIM) (34.6%), systemic lupus erythematosus (SLE) (11.9%), and rheumatoid arthritis (RA) (11.9%). (Fig. 2). Most patients were given glucocorticosteroids (142, 89.3%) and/or immunosuppressants (112, 70.4%) for CTD-ILD treatment before PJP. At the onset of PJP, the average corticosteroid dosage of the patients was 40 (25, 55) mg/d prednisolone equivalent. The interval between the onset and diagnosis of PJP was 8 (4, 16) days. The main clinical manifestations of PJP in CTD-ILD patients were dyspnea (144, 90.6%), cough (133, 83.6%), fever (130, 81.8%), and expectoration (116, 73.0%). The common chest CT manifestations in CTD-ILD-PJP patients are listed in supplementary Table 1.

A total of 120 (75.5%) CTD-ILD-PJP patients experienced respiratory failure, and the average oxygenation index (PaO_2_/FiO_2_ ratio) was 178 (115, 288) mmHg. Moreover, 87 (54.7%) patients were transferred to intensive care unit (ICU) wards, and 75 (47.2%) patients received invasive mechanical ventilation support. After the diagnosis of PJP, almost all CTD-ILD-PJP patients received trimethoprim-sulfamethoxazole (TMPco) as primary treatment (158/159, 99.4%), and most patients (128, 80.5%) were prescribed 15 mg/kg/d TMPco. Approximately one-third of our patients (48, 30.2%) were also prescribed combined second-line anti-PJP medications. Ultimately, there were 81 (50.9%) survival patients and 78 (49.1%) non-survival patients. The main causes of death included severe PJP infection-associated respiratory failure, concurrent severe mixed pulmonary infectious diseases, and progression of underlying ILD after the PJP episode.

### Differences in the characteristics and prognoses of CTD-PJP patients with and without ILD

The differences among the CTD-ILD-PJP patients and CTD-non-ILD-PJP patients are listed in Table 1. Compared with those in the CTD-non-ILD-PJP group, the patients in the CTD-ILD-PJP group were older (57.4 ± 14.8 years vs. 46.7 ± 16.9 years, *p* < 0.001). There were more systemic vasculitidese and IIM patients in the CTD-ILD-PJP group and more systemic lupus erythematosus patients in the CTD-non-ILD-PJP group (*p* < 0.001). In terms of primary disease treatment before PJP infection, more patients were treated with cyclophosphamide in the CTD-non-ILD-PJP group (39.0% vs. 21.3%, *p* = 0.004). Furthermore, both the interval between glucocorticosteroid treatment and PJP infection [75.0 (36.0, 197.0) days vs. 99.0 (69.0, 945.0) days, *p* < 0.001] and between immunosuppressant treatment and PJP infection [69.5 (40.5, 177.3) days vs. 90.5 (57.0, 767.5) days, *p* = 0.028] were shorter in the CTD-ILD-PJP group than in the CTD-non-ILD-PJP group.

**Table 1:** The characteristics between CTD-ILD-PJP group and CTD-non-ILD-PJP group

Variables	CTD-ILD-PJP group (n = 159)	CTD-non-ILD-PJP group (n = 89)	t / Z /χ^2^	*P* Value
Baseline characteristics				
Age (years) ^a^	57.4 ± 14.8	46.7 ± 16.9	−5.027	** < 0.001**
Male ^b^	62 (39.0)	26 (29.2)	2.384	0.123
Underlying CTDs ^b^			111.001	** < 0.001**
Systemic vasculitides	57 (35.8)	6 (6.7)		
IIM	55 (34.6)	3 (3.4)		
SLE	19 (11.9)	38 (42.7)		
RA	19 (11.9)	4 (4.5)		
Other CTDs	9 (5.7)	38 (42.7)		
Hyperglycemia or diabetes mellitus^b^	92 (57.9)	49 (55.1)	0.183	0.669
The interval between onset and diagnosis of PJP (days) ^c^	8.0 (4.0, 16.0)	8.0 (4.0, 14.0)	−0.083	0.934
Primary treatment for CTDs				
Glucocorticosteroids ^b^	142 (89.3)	83 (93.3)	1.058	0.304
The interval between glucocorticosteroids treatment and PJP (days) ^c^	75.0 (36.0, 197.0)	99.0 (69.0, 945.0)	−3.837	** < 0.001**
Prednisone dosage (mg/d) ^c^	40.0 (25.0, 55.0)	40.0 (25.0, 55.0)	−0.437	0.662
Immunosuppressants ^b^	112 (70.4)	68 (76.4)	1.020	0.313
CTX ^b^	62 (39.0)	19 (21.3)	8.078	**0.004**
MMF ^b^	18 (11.3)	14 (15.7)	0.987	0.320
MTX ^b^	17 (10.7)	14 (15.7)	1.324	0.250
The interval between immunosuppressants treatment and PJP (days) ^c^	69.5 (40.5, 177.3)	90.5 (57.0, 767.5)	−2.200	**0.028**
Infection status				
PaO2/FiO2 (mmHg)^c^	178.0 (115.0, 288.0)	206.0 (127.0, 310.0)	−1.127	0.260
Respiratory failure ^b^	120 (75.5)	62 (69.7)	0.986	0.321
ICU admission ^b^	87 (54.7)	46 (51.7)	0.211	0.646
Invasive ventilation ^b^	75 (47.2)	35 (39.3)	0.233	1.422
Pneumomediastinum ^b^	26 (16.4)	6 (6.7)	4.690	**0.030**
CMV viremia ^b^	84 (52.8)	48 (53.9)	0.028	0.867
Oral candidiasis infection ^b^	18 (11.3)	11 (12.4)	0.060	0.807
Mycobacteria infection^b^	4 (2.5)	4 (4.5)	0.222	0.637
Nocardia infection^b^	4 (2.5)	0 (0.0)	0.966	0.326
Aspergilli infection^b^	28 (17.6)	10 (11.2)	1.787	0.181
HAP ^b^	48 (30.2)	23 (25.8)	0.527	0.468
Chest CT features				
Bilateral ^b^	155 (97.5)	88 (98.9)	0.077	0.782
GGOs ^b^	125 (78.6)	76 (85.4)	1.706	0.191
Patches ^b^	124 (78.0)	64 (71.9)	1.149	0.284
Consolidation ^b^	48 (30.2)	34 (38.2)	1.656	0.198
Nodular ^b^	47 (29.6)	36 (40.4)	3.039	0.081
Treatment for PJP				
Therapeutic dose TMPco ^b^	128 (80.5)	69 (77.5)	0.309	0.578
Combination with second-line treatment ^b^	48 (30.2)	25 (28.1)	0.121	0.728
Prognosis				
All-cause mortality ^b^	78 (49.1)	30 (33.7)	5.468	**0.019**

^a^ mean ± SD, ^b^ n (%), ^c^ median (IQR). Bold values mean *p* value < 0.05. CTD, connective tissue diseases; IIM, idiopathic inflammatory myopathy; SLE, systemic lupus erythematosus; RA, rheumatoid arthritis; ILD, interstitial lung disease; PJP, *Pneumocystis jirovecii* pneumonia; CTX, cyclophosphamide; MMF, mycophenolate mofetil; MTX, methotrexate; CMV, cytomegalovirus; HAP, hospital-acquired pneumonia; GGO, ground-glass opacities; TMPco, trimethoprim-sulfamethoxazole; ICU, intensive care unit

There was no significant difference in the PaO2/FiO2 ratio, ICU admission or invasive ventilation between the two groups. The incidence rates of complicated coinfection diseases were also similar between the two groups. However, after PJP infection, more patients in the CTD-ILD-PJP group developed pneumomediastinum (16.4% vs. 6.7%, *p* = 0.030). Furthermore, the differences in the results of laboratory examinations between the two groups of patients are described below (see supplementary Table 2). Compared with those in the CTD-non-ILD-PJP group, patients in the CTD-ILD-PJP group had higher white blood cell count [7.7 (5.6, 10.9) × 10^9^/L vs. 6.4 (4.1, 8.9) × 10^9^/L, *p* = 0.030], neutrophil count [6.6 (4.8, 9.4) × 10^9^/L vs. 5.4 (3.4, 7.9) × 10^9^/L, *p* = 0.002], platelet (PLT) [183.0 (129.0, 226.0) × 10^9^/L vs. 128.0 (94.0, 213.5) × 10^9^/L, *p* = 0.003], immunoglobulin A (1.9 ± 1.0 g/L vs. 1.6 ± 1.2 g/L, *p* = 0.043), and CD4^+^T/CD8^+^ T-cell [0.9 (0.4, 1.8) vs. 0.6 (0.4, 1.0), *p* < 0.015]. In addition, there were more patients who were 1,3-β-D-glucan-positive in the CTD-non-ILD-PJP group (78.3% vs. 89.2%, *p* = 0.040).

**Fig. 3:**
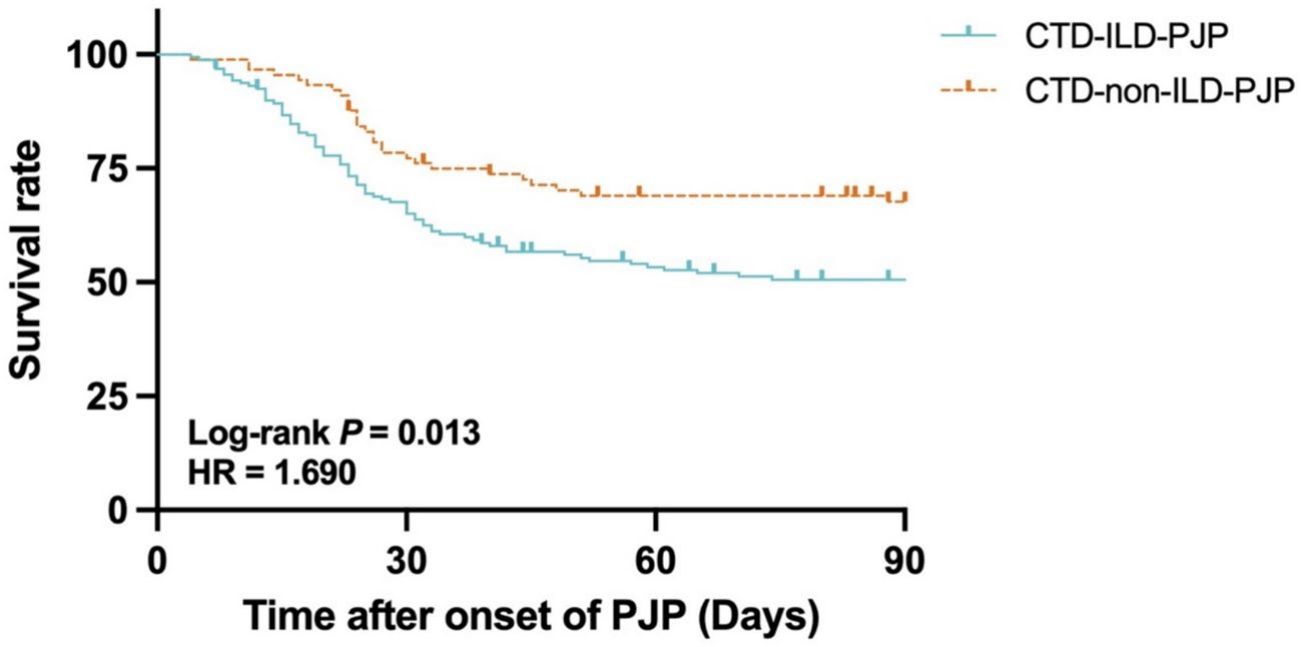
Kaplan–Meier analysis of CTD-PJP patients with ILD and without ILD. CTD, connective tissue diseases; PJP, *Pneumocystis jirovecii* pneumonia; ILD, interstitial lung disease

The all-cause mortality of patients was significantly greater in the CTD-ILD-PJP group than in the CTD-non-ILD-PJP group (49.1% vs. 33.7%, *p* = 0.019). Kaplan–Meier analysis (Fig. 3) also revealed that CTD-PJP patients with ILD had significantly worse outcomes than those without ILD.

### Differences between the survival and non-survival groups of CTD-ILD-PJP patients

We compared the differences in the clinical outcomes between the survival and non-survival CTD-ILD-PJP patients, the results of which are listed in Table 2. First, the distribution of the underlying CTDs differed between the two groups (*p* = 0.007). There were more IIM patients with ILD-PJP (46.2%) in non-survival group than in the survival group, while there were more systemic vasculitidese patients with ILD-PJP (38.3%) in the survival group. Additionally, a similar proportion of patients in the two groups received glucocorticosteroid administration before PJP infection, and there was also no significant difference in the dosage of glucocorticosteroids. The interval between the administration of glucocorticosteroids was shorter [57.0 (32.0, 135.3) days vs. 86.0 (39.0, 239.0) days, *p* = 0.043] in the non-survival group. However, more patients received methotrexate (MTX) (17.3% vs. 3.8%, *p* = 0.009) in the survival group.

**Table 2:** The clinical characteristics between survival group and non-survival group

Variables	Survival group (n = 81)	Non-survival group (n = 78)	t / Z /χ^2^	*P* Value
Baseline characteristics				
Age (years) ^c^	58.0 (51.5, 67.0)	61.0 (50.8, 69.3)	−0.829	0.407
Male ^b^	27 (33.3)	35 (44.9)	2.224	0.147
Underlying CTDs ^b^			13.717	**0.007**
Systemic vasculitides	31 (38.3)	26 (33.3)		
IIM	19 (23.5)	36 (46.2)		
SLE	10 (12.3)	9 (11.5)		
RA	13 (16.0)	6 (7.7)		
Other CTDs	8 (9.9)	1 (1.3)		
Hyperglycemia or diabetes mellitus^b^	41 (50.6))	51 (65.4)	3.554	0.077
The interval between onset and diagnosis of PJP (days) ^c^	8.0 (4.0, 17.0)	7.0 (4.0, 15.3)	−0.124	0.901
Primary treatment for CTDs				
Glucocorticosteroids ^b^	76 (93.8)	66 (84.6)	3.531	0.074
The interval between glucocorticosteroids treatment and PJP (days) ^c^	86.0 (39.0, 239.0)	57.0 (32.0, 135.3)	−2.025	**0.043**
Steroid pulse therapy ^b^	13 (16.0)	13 (16.7)	0.011	1.000
Current prednisone dosage (mg/d) ^c^	35.0 (22.5, 50.0)	40.0 (30.0, 60.0)	−1.352	0.176
Cumulative prednisone dosage before PJP				
Prednisone dosage > 15 mg/d within 4 weeks ^b^	63 (77.8)	55 (70.5)	1.096	0.295
Prednisone dosage in 1 year ^c^	2850.0 (1807.5, 5017.5)	2410.0 (460.0, 4357.5)	−1.387	0.165
Immunosuppressants ^b^	59 (72.8)	53 (67.9)	0.456	0.602
CTX ^b^	29 (35.8)	33 (42.3)	0.707	0.420
MMF ^b^	10 (12.3)	8 (10.3)	0.173	0.804
MTX ^b^	14 (17.3)	3 (3.8)	7.514	**0.009**
The interval between immunosuppressants treatment and PJP (days) ^c^	62.0 (27.5, 126.8)	80.5 (48.3, 237.0)	−1.710	0.087
Infection status				
PaO_2_/FiO_2_ (mmHg) ^c^	243.0 (182.0, 329.0)	126.0 (89.0, 169.0)	−6.665	** < 0.001**
Pneumomediastinum ^b^	5 (6.2)	21 (26.9)	12.508	** < 0.001**
CMV viremia ^b^	40 (49.4)	44 (56.4)	0.787	0.428
Oral candidiasis infection ^b^	7 (8.6)	11 (14.1)	1.180	0.323
Mycobacteria infection^b^	3 (3.7)	1 (1.3)	0.950	0.620
Nocardia infection^b^	1 (1.2)	3 (3.8)	1.105	0.361
Aspergilli infection^b^	7 (8.6)	21 (26.9)	9.153	**0.003**
HAP ^b^	17 (21.0)	31 (39.7)	6.633	**0.015**
Chest CT features				
Bilateral ^b^	79 (97.5)	76 (97.4)	0.001	1.000
GGOs ^b^	65 (80.2)	60 (76.9)	0.261	0.700
Patches ^b^	68 (84.0)	56 (71.8)	3.420	0.085
Consolidation ^b^	23 (28.4)	25 (32.1)	0.252	0.730
Nodular ^b^	33 (40.7)	14 (17.9)	9.913	**0.002**
Reticular ^b^	27 (33.3)	27 (34.6)	0.029	0.865
Honeycomb ^b^	4 (4.9)	11 (14.1)	3.906	**0.048**
Treatment for PJP				
Combination with second-line treatment ^b^	18 (22.2)	30 (38.5)	4.972	**0.038**

^a^ mean ± SD, ^b^ n (%), ^c^ median (IQR). Bold values mean *p* value < 0.05. CTD, connective tissue diseases; IIM, idiopathic inflammatory myopathy; SLE, systemic lupus erythematosus; RA, rheumatoid arthritis; ILD, interstitial lung disease; PJP, *Pneumocystis jirovecii* pneumonia; CTX, cyclophosphamide; MMF, mycophenolate mofetil; MTX, methotrexate; CMV, cytomegalovirus; HAP, hospital-acquired pneumonia; GGO, ground-glass opacities; TMPco, trimethoprim-sulfamethoxazole

The PaO_2_/FiO_2_ ratio in the non-survival group was much lower than that in the survival group [126.0 (89.0, 169.0) mmHg vs. 243.0 (182.0, 329.0) mmHg,* p* < 0.001]. Patients in the non-survival group were more likely to suffer from pneumomediastinum (26.9% vs. 6.2%, *p* < 0.001), HAP (39.7% vs. 21.0%, *p* = 0.015) and aspergilli infection (26.9% vs. 8.6%, *p* = 0.003). The chest CT features of the two groups also differed: nodules were more common in the survival group (17.9% vs. 40.7%, *p* = 0.002), while honeycomb was more common in the non-survival group (14.1% vs. 4.9%, *p* = 0.048). The differences in laboratory test results between the two groups of CTD-ILD-PJP patients were as follows (see supplementary Table 3). Patients in the non-survival group had significantly lower peripheral lymphocyte counts (0.5 ± 0.5 × 10^9^/L vs. 1.1 ± 1.3 × 10^9^/L, *p* < 0.001), PLT counts (159.6 ± 94.2 × 10^9^/L vs. 204.3 ± 87.9 × 10^9^/L, *p* = 0.002), minimal albumin (Alb) (21.5 ± 4.3 g/L vs. 27.4 ± 4.4 g/L, *p* < 0.001), and complement 3 (0.9 ± 0.3 g/L vs. 1.0 ± 0.3 g/L, *p* = 0.031). In the peripheral lymphocyte subset analysis, the natural killer (NK) cell count [16.0 (7.0, 49.3)/μL vs. 46.0 (17.0, 98.0)/μL, *p* < 0.001], T-cell count [262.5 (131.5, 424.8)/μL vs. 439.0 (278.5, 715.0)/μL, *p* < 0.001], and CD4^+^ T-cell count [96.0 (46.3, 181.5)/μL vs. 159.0 (79.0, 317.5)/μL, *p* < 0.001] were also lower in the non-survival group than in the survival group. Additionally, patients in the non-survival group had higher levels of lactate dehydrogenase [596.0 (418.8, 861.0) U/L vs. 422.0 (319.5, 545.5) U/L, *p* < 0.001], ferritin [1223.5 (650.8, 2292.0) μg/L vs. 452.0 (306.0, 967.0) μg/L, *p* < 0.001] and D-dimer [2.8 (1.1, 6.9) mg/L vs. 1.1 (0.5, 2.9) mg/L, *p* < 0.001].

With respect to the treatment of PJP, patients in the non-survival group were more likely to be prescribed combination second-line antibiotic treatments (38.5% vs. 22.2%, *p* = 0.038).

### Prognostic analysis for CTD-ILD-PJP patients

**Fig. 4:**
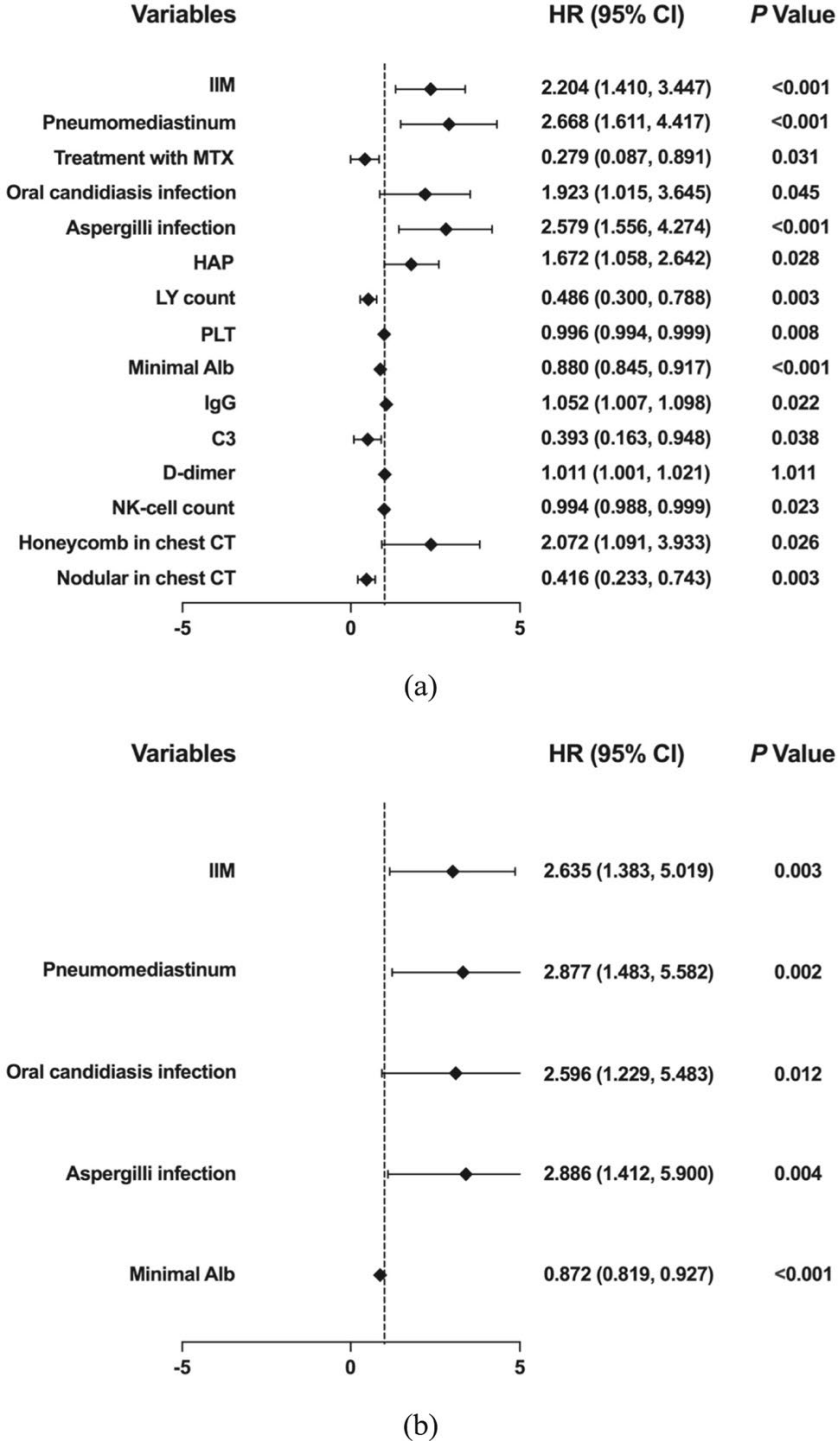
Univariable (**a**) and multivariable (**b**) Cox regression analysis of survival associated risk factors for patients with CTD-ILD-PJP. IIM, idiopathic inflammatory myopathy; MTX, methotrexate; HAP, hospital-acquired pneumonia; LY, lymphocyte; PLT, platelet; Alb, albumin; Ig, immunoglobulin; C3, complement 3

We included the following variables in univariate Cox regression analysis: Age, gender, underlying CTDs, the interval between onset and diagnosis of PJP, treatment status of glucocorticosteroids or immunosuppressants, pneumomediastinum, co-infection, chest CT findings and treatment for PJP. The results (Fig. 4a) revealed that IIM, pneumomediastinum, oral candidiasis infection, aspergilli infection, HAP, and honeycomb in the chest CT images were associated with poor prognosis in CTD-ILD-PJP patients. Additionally, lower peripheral lymphocyte count, PLT, minimal Alb, immunoglobulin G, complement 3, and NK cell count and higher D-dimer were associated with mortality in CTD-ILD-PJP patients. However, treatment with MTX and nodular in chest CT might be associated with survival. Finally, multivariate Cox regression analysis (Fig. 4b) revealed that IIM (HR = 2.635, χ^2^ = 8.679, 95% CI: 1.383–5.019), pneumomediastinum (HR = 2.877, χ^2^ = 9.768, 95% CI: 1.483–5.582), oral candidiasis infection (HR = 2.596, χ^2^ = 6.249, 95% CI: 1.229–5.483), aspergilli infection (HR = 2.886, χ^2^ = 8.438, 95% CI: 1.412–5.900), and lower minimal Alb (HR = 0.872, χ^2^ = 18.983, 95% CI: 0.819–0.927) were independent risk factors associated with poor survival in patients with CTD-ILD-PJP.

## Discussion

We focused on the characteristics and prognosis of PJP infection in CTD-ILD patients. Our retrospective study revealed that the mortality rate of CTD-ILD-PJP patients was high. The prognosis of CTD-ILD-PJP patients was worse than that of CTD-PJP patients without ILD. IIM, pneumomediastinum, oral candidiasis infection, aspergilli infection, and lower minimal Alb were independent risk factors associated with poor survival in CTD-ILD-PJP patients.

Previous studies have shown that ILD increases the risk of PJP infection [12–15], which coincides with our findings. In our CTD-PJP cohort, most patients had ILD (64.1%). Our study also revealed that CTD-ILD-PJP patients were mainly middle-aged females. The underlying CTDs of our CTD-ILD-PJP patients were mainly systemic vasculitis, IIM, systemic lupus erythematosus and rheumatoid arthritis. Additionally, most CTD-ILD patients were treated with glucocorticosteroids and/or immunosuppressants before PJP infection. The average corticosteroid dosage used to treat the patients was 40 (25, 55) mg/d prednisolone equivalent, and the average interval between glucocorticosteroid treatment and PJP was 75.0 (36.0, 197.0) days. A retrospective study also revealed that the mean duration of glucocorticoid therapy was 71 days and that the mean daily dose of prednisolone was 37 mg in ILD-PJP patients [16]. After PJP infection, most patients experienced respiratory failure. Furthermore, approximately half of the patients received intensive care in the ICU and invasive mechanical ventilation support. The main causes of death included infection-associated respiratory failure and progression of underlying ILD after PJP infection.

Our results revealed that the prognosis of CTD-ILD-PJP patients was significantly worse than that of CTD-non-ILD-PJP patients, which was consistent with the findings of previous studies [4, 17, 18]. Ishikawa Y et al. conducted a retrospective study including 333 CTD-PJP patients and reported that ILD was associated with poor prognosis (HR = 1.65, *p* = 0.012, 95% CI: 1.12–2.42) [4]. According to our findings, the severity of underlying CTDs, PJP infection status and laboratory results in CTD-ILD-PJP patients were not significantly different than those in patients without ILD, which may suggest that structural damage to the lungs has a greater impact on the prognosis of CTD-PJP patients. The increased mortality of CTD-ILD-PJP patients may be due to several reasons. First, glucocorticosteroids and immunosuppressants, which can impair the immune function of patients and reduce the CD4^+^T cell count, are commonly used to treat CTD-ILD-PJP patients. Second, there is a high rate of PJ colonization in ILD patients in the damaged lung [19]. Finally, both the respiratory symptoms (such as dry cough, dyspnea, hypoxia, etc.) and manifestations on chest HRCT (such as diffuse ground-glass opacities, consolidations, architectural distortions, etc.) of PJP infection are similar to those of ILD, which increases the difficulty of diagnosing PJP in ILD patients [20–22]. Interestingly, we also found that the positive rate of the 1,3-β-D-glucan test in CTD-ILD-PJP patients was significantly lower than that in CTD-non-ILD-PJP patients. Yuan Huang et al. reported that the 1,3-β-D-glucan test had insufficient diagnostic efficacy for PJP in patients with lung cancer and ILD [23]. The reason was not clear, probably because of insufficient regional or diffuse parenchymal blood supply. Therefore, we cannot ignore CTD-ILD patients with new respiratory symptoms and imaging lesions consistent with PJP infection but who are negative for 1,3-β-D-glucan.

Previous studies have shown that prior glucocorticoid therapy, decreased CD4^+^ T-cell count, and increased 1,3-β-D-glucan level are risk factors for PJP in patients with CTD-ILD [6, 24]. However, few studies have analyzed the factors associated with CTD-ILD-PJP patient prognosis. We compared the survival and non-survival patients, and found that IIM was an independent factor for poor CTD-ILD-PJP patient prognosis. Most likely, because a special subtype of IIM, anti-MDA5-antibody-positive (anti-MDA5^+^) dermatomyositis, is usually associated with rapidly progressive ILD. Our previous study showed that compared to the survival group, there were more patients with anti-MDA5^+^ in non-survival group (81.5% vs. 45.0%, *p* = 0.009) and anti-MDA5^+^ was one of the independent risk factors for IIM-PJP patients (HR = 3.54, *p* = 0.016, 95% CI: 1.27–9.89) [25]. Moreover, anti-MDA5^+^ dermatomyositis patients often receive treatment with high doses of glucocorticoids combined with immunosuppressants, which leads to increased susceptibility to opportunistic infections such as PJP and a poor prognosis [26–29]. Huang L et al. reported that patients with anti-MDA5^+^ dermatomyositis did not seem to benefit from timely administration of anti-PJP treatment within six days after PJP infection, unlike patients with other rheumatic diseases (*p* = 0.327) [30].

In addition, pneumomediastinum is associated with poor prognosis in CTD-ILD-PJP patients. Pneumomediastinum is a condition in which air is present in the lung interstitium after the rupture of the pulmonary alveolus and along the vascular bundle to the hilum of the lung and mediastinum. The incidence of pneumomediastinum in PJP patients can reach as high as 36.4% [31]. ILD-PJP patients are more likely to develop pneumomediastinum due to pulmonary interstitial changes.

Our study also revealed that oral candidiasis infection and aspergilli infection were associated with mortality in CTD-ILD-PJP patients, which suggested impaired immune function in these patients. Sanadhya et al. reported a definite relationship between the occurrence of oral lesions and opportunistic infections among HIV-infected patients [32]. For immunosuppressed people, oral leukoplakia may be the first clinical sign of opportunistic infections such as PJP. Moreover, oral candidiasis infection can be detected early through detailed physical examination, which could be used as a noninvasive and low-cost predictor.

There is no consensus on a PJP prevention protocol for patients with CTDs or ILDs. However, considering the high mortality of CTD-ILD-PJP patients, it is still necessary to selectively prevent PJP in high-risk patients. TMPco, which is currently recommended as a first-line PJP prevention and treatment medication, has been shown to be effective. Recent studies recommended that non-HIV immunocompromised patients receiving long-term treatment with glucocorticosteroids (prednisone doses exceeding 20 mg/day) should receive preventative treatment with TMPco [33]. In clinical practice, physicians should pay more attention to CTD-ILD patients treated with corticosteroids combined with immunosuppressants, especially IIM patients. After contraindications are excluded, prophylactic TMPco treatment can be actively administered to reduce the incidence and mortality of PJP in CTD-ILD patients.

There are several limitations in our study. First, this was a retrospective investigation, and all patients were hospitalized at a famous tertiary medical center, which could lead to selection bias. Second, there were no detailed analyses of the location and severity of lung lesions in patients with ILD, which could impact on the prognosis of PJP patients. Third, lymphocyte subset analysis was not performed after the patients recovered from PJP. Lastly, the management protocols for CTD-ILD, such as cumulative corticosteroid dosage, and the status of PJP prophylaxis could not be collected completely.

## Conclusion

CTD-ILD-PJP patients were mainly middle-aged females and had higher mortality rates than CTD-PJP patients without ILD. IIM, pneumomediastinum, oral candidiasis infection, aspergilli infection, and lower minimal Alb were independent risk factors associated with poor survival in CTD-ILD-PJP patients.

## Supplementary information

Below is the link to the electronic supplementary material.

ESM 1 (DOCX 62.4 KB)

## Data Availability

Upon reasonable request, the corresponding author will provide the data.
