# Supplementary material for: Characteristics and prognostic analysis of Pneumocystis jirovecii pneumonia in connective tissue diseases patients with interstitial lung disease: a retrospective study
Source: Clin Rheumatol. 2025 Mar 6;44(4):1653–63. doi: 10.1007/s10067-025-07392-1 (PMC11993478; doi:10.1007/s10067-025-07392-1)
Supplement: Supplementary file 1 — (DOCX 62.4 KB) [file 10067_2025_7392_MOESM1_ESM.docx]

**Title**: Characteristics and prognostic analysis of *Pneumocystis jirovecii* pneumonia in connective tissue diseases patients with interstitial lung disease: a retrospective study

**Journal name**: Clinical Rheumatology

***Author names****: Yujie Shi, Ruxuan Chen, Hongli Sun, Kai Xu, Mengqi Wang, Zhiyi Li, Chi Shao, Hui Huang*

***Correspondence****: Hui Huang, Department of Pulmonary and Critical Care Medicine, Peking Union Medical College Hospital, Chinese Academy of Medical Sciences & Peking Union Medical College, No.1 Shuaifuyuan St, Dongcheng District, Beijing, China, 100730. Email: pumchhh@126.com*

**Table S1 The chest CT features in CTD-ILD-PJP patients.**

| **Chest CT features** | **CTD-ILD-PJP patients**  **(n = 159)** |
| --- | --- |
| Bilateral ^b^ | 155 (97.5) |
| Sub-pleural distribution ^b^ | 78 (49.1) |
| Peri-bronchial distribution ^b^ | 24 (15.1) |
| GGOs ^b^ | 125 (78.6) |
| Patches ^b^ | 124 (78.0) |
| Consolidation ^b^ | 48 (30.2) |
| Reticular ^b^ | 54 (34.0) |
| Honeycomb ^b^ | 15 (9.4) |
| Septal thickening ^b^ | 47 (29.6) |
| Nodular ^b^ | 47 (29.6) |
| Pleural thickening ^b^ | 117 (73.6) |
| Pleural effusion ^b^ | 51 (32.1) |
| Pericardial effusion ^b^ | 35 (22.0) |
| Lymphadenopathy ^b^ | 83 (52.2) |

^b^ n (%). CTD, connective tissue diseases; ILD, interstitial lung disease; PJP, *Pneumocystis* *jirovecii* pneumonia; GGO, ground-glass opacities.

**Table S2 The laboratory examination between CTD-ILD-PJP group and CTD-non-ILD-PJP group.**

| **Variables** | **CTD-ILD-PJP group**  **(n = 159)** | **CTD-non-ILD-PJP group**  **(n = 89)** | **t / Z /χ^2^** | ***P* Value** |
| --- | --- | --- | --- | --- |
| WBC (×10^9^/L) ^c^ | 7.7 (5.6, 10.9) | 6.4 (4.1, 8.9) | -2.842 | **0.030** |
| NEUT count (×10^9^/L) ^c^ | 6.6 (4.8, 9.4) | 5.4 (3.4, 7.9) | -3.069 | **0.002** |
| LY count (×10^9^/L) ^c^ | 0.5 (0.3, 0.9) | 0.6 (0.3, 0.9) | -0.400 | 0.689 |
| HGB (g/L) ^a^ | 112.2 ± 23.9 | 102.4 ± 21.8 | -3.225 | **0.001** |
| PLT (×10^9^/L) ^c^ | 183.0 (129.0, 226.0) | 128.0 (94.0, 213.5) | -2.983 | **0.003** |
| Minimal Alb (g/L) ^a^ | 24.5 ± 5.3 | 24.3 ± 5.6 | -0.338 | 0.736 |
| Serum Cr (μmol/L) ^c^ | 70.0 (52.0, 118.0) | 72.5 (50.0, 140.5) | -0.315 | 0.753 |
| Urea (mmol/L) ^c^ | 7.4 (5.4, 11.7) | 7.9 (5.2, 11.0) | -0.031 | 0.975 |
| IgG (g/L) ^c^ | 8.1 (5.8, 11.8) | 7.3 (4.9, 10.8) | -1.287 | 0.198 |
| IgA (g/L) ^a^ | 1.9 ± 1.0 | 1.6 ± 1.2 | -2.035 | **0.043** |
| IgM (g/L) ^c^ | 0.7 (0.4, 1.3) | 0.6 (0.3, 1.3) | -1.169 | 0.243 |
| C3 (g/L) ^a^ | 1.0 ± 0.3 | 1.0 ± 0.3 | -0.233 | 0.816 |
| C4 (g/L) ^c^ | 0.2 (0.2, 0.3) | 0.2 (0.1, 0.3) | -1.623 | 0.104 |
| LDH (U/L) ^c^ | 483.0 (342.5, 665.0) | 533.5 (380.5, 767.3) | -1.558 | 0.119 |
| hsCRP (mg/L) ^c^ | 37.6 (11.9, 95.7) | 30.5 (6.0, 95.0) | -0.563 | 0.574 |
| ESR (mm/h) ^a^ | 54.0 ± 33.5 | 48.3 ± 33.4 | -1.209 | 0.228 |
| Fer (μg/L) ^c^ | 792.0 (402.0, 1527.0) | 840.5 (270.8, 1542.0) | -0.593 | 0.553 |
| D-Dimer (mg/L) ^c^ | 1.7 (0.7, 5.1) | 1.3 (0.5, 5.2) | -0.994 | 0.320 |
| Fbg (g/L) ^c^ | 3.8 (2.8, 5.0) | 3.8 (2.9, 4.9) | -0.212 | 0.832 |
| 1,3-β-D-glucan ^b^ | 108 (78.3) | 74 (89.2) | 4.234 | **0.040** |
| D(A-a)O_2_ (mmHg) ^c^ | 45.2 (28.9, 56.9) | 48.3 (30.9, 58.1) | -0.594 | 0.552 |
| Peripheral lymphocyte subset analysis | |  |  |  |
| B cell count (/μL) ^c^ | 37.0 (10.8, 112.0) | 37.0 (4.0, 101.3) | -0.669 | 0.504 |
| NK cell count (/μL) ^c^ | 31.0 (10.0, 79.5) | 33.5 (12.0, 73.8) | -0.277 | 0.782 |
| T cell count (/μL) ^c^ | 341.0 (185.5, 548.5) | 389.0 (159.8, 668.0) | -0.778 | 0.437 |
| CD4^+^T cell count (/μL) ^c^ | 132.0 (61.0, 256.0) | 114.0 (59.3, 268.8) | -0.620 | 0.535 |
| CD8^+^T cell count (/μL) ^c^ | 161.0 (72.0, 277.5) | 202.0 (90.8, 464.8) | -1.857 | 0.063 |
| CD4^+^T / CD8^+^T ^c^ | 0.9 (0.4, 1.8) | 0.6 (0.4, 1.0) | -2.437 | **0.015** |

^a^ mean ± SD, ^b^ n (%), ^c^ median (IQR). Bold values mean *p* value < 0.05. WBC, white blood cell; NEUT, neutrophil; LY, lymphocyte; HGB, hemoglobin; PLT, platelet; Alb, albumin; Cr, creatinine; Ig, immunoglobulin; C, complement; LDH, lactate dehydrogenase; hsCRP, high-sensitivity C-reactive protein; ESR, erythrocyte sedimentation rate; Fer, ferritin; Fbg, Fibrinogen.

**Table S3 The laboratory examination between survival group and non-survival group.**

| **Variables** | **Survival group**  **(n = 81)** | **Non-survival group**  **(n = 78)** | **t / Z /χ^2^** | ***P* Value** |
| --- | --- | --- | --- | --- |
| WBC (×10^9^/L) ^a^ | 8.5 ± 4.1 | 8.3 ± 3.7 | 0.310 | 0.757 |
| NEUT count (×10^9^/L) ^a^ | 7.2 ± 3.7 | 7.5 ± 3.4 | -0.610 | 0.543 |
| LY count (×10^9^/L) ^a^ | 1.1 ± 1.3 | 0.5 ± 0.5 | 3.491 | **< 0.001** |
| HGB (g/L) ^c^ | 115.0 (103.0, 133.0) | 109.0 (93.5, 126.8) | -1.599 | 0.110 |
| PLT (×10^9^/L) ^a^ | 204.3 ± 87.9 | 159.6 ± 94.2 | 3.096 | **0.002** |
| Minimal Alb (g/L) ^a^ | 27.4 ± 4.4 | 21.5 ± 4.3 | 8.606 | **< 0.001** |
| Serum Cr (μmol/L) ^c^ | 70.0 (54.0, 101.0) | 69.0 (48.8, 121.0) | -0.362 | 0.717 |
| Urea (mmol/L) ^c^ | 7.2 (5.4, 9.7) | 8.0 (5.4, 13.1) | -0.815 | 0.415 |
| IgG (g/L) ^c^ | 7.5 (5.8, 10.8) | 8.8 (5.7, 14.0) | -1.033 | 0.301 |
| IgA (g/L) ^a^ | 1.8 ± 1.1 | 1.9 ± 0.9 | -0.714 | 0.477 |
| IgM (g/L) ^c^ | 0.7 (0.4, 1.5) | 0.7 (0.5, 1.3) | -0.025 | 0.980 |
| C3 (g/L) ^a^ | 1.0 ± 0.3 | 0.9 ± 0.3 | 2.180 | **0.031** |
| C4 (g/L) ^a^ | 0.2 ± 0.1 | 0.2 ± 0.1 | 1.415 | 0.159 |
| LDH (U/L) ^c^ | 422.0 (319.5, 545.5) | 596.0 (418.8, 861.0) | -4.432 | **< 0.001** |
| hsCRP (mg/L) ^c^ | 37.2 (11.4, 86.9) | 38.0 (12.5, 104.6) | -0.656 | 0.512 |
| ESR (mm/h) ^a^ | 51.1 ± 29.5 | 57.0 ± 37.2 | -1.077 | 0.283 |
| Fer (μg/L) ^c^ | 452.0 (306.0, 967.0) | 1223.5 (650.8, 2292.0) | -4.140 | **< 0.001** |
| D-Dimer (mg/L) ^c^ | 1.1 (0.5, 2.9) | 2.8 (1.1, 6.9) | -4.141 | **< 0.001** |
| Fbg (g/L) ^a^ | 4.2 ± 1.7 | 3.9 ± 1.8 | 1.126 | 0.262 |
| 1,3-β-D-glucan ^b^ | 55 (75.3) | 53 (81.5) | 0.776 | 0.378 |
| D(A-a)O_2_ (mmHg) ^c^ | 45.0 (24.4, 55.2) | 47.6 (33.0,57.7) | -1.201 | 0.230 |
| Peripheral lymphocyte subset analysis | |  |  |  |
| B cell count (/μL) ^c^ | 41.0 (15.0, 117.0) | 33.0 (9.0, 96.0) | -0.785 | 0.432 |
| NK cell count (/μL) ^c^ | 47.5 (18.8, 103.0) | 17.0 (6.0, 49.0) | -3.112 | **0.002** |
| T cell count (/μL) ^c^ | 397.0 (275.0, 675.0) | 283.5 (136.3, 429.0) | -3.074 | **0.002** |
| CD4^+^T cell count (/μL) ^c^ | 163.0 (76.0, 263.0) | 101.5 (43.3, 212.0) | -2.282 | **0.023** |
| CD8^+^T cell count (/μL) ^a^ | 260.1 ± 243.2 | 199.6 ± 323.0 | 1.202 | 0.232 |
| CD4^+^T / CD8^+^T ^c^ | 1.0 (0.4, 1.9) | 0.8 (0.5, 1.6) | -0.214 | 0.830 |

^a^ mean ± SD, ^b^ n (%), ^c^ median (IQR). Bold values mean *p* value < 0.05. WBC, white blood cell; NEUT, neutrophil; LY, lymphocyte; HGB, hemoglobin; PLT, platelet; Alb, albumin; Cr, creatinine; Ig, immunoglobulin; C, complement; LDH, lactate dehydrogenase; hsCRP, high-sensitivity C-reactive protein; ESR, erythrocyte sedimentation rate; Fer, ferritin; Fbg, Fibrinogen.
